# First case of urban leishmaniasis in the Campeche State, Mexico

**DOI:** 10.1590/S1678-9946202466058

**Published:** 2024-10-11

**Authors:** Selene Blum-Domínguez, Daniel Sokani Sánchez-Montes, Ingeborg Becker, Rolando García-Martínez, Paulino Tamay-Segovia

**Affiliations:** 1 Universidad Autónoma de Campeche, Centro de Investigaciones Biomédicas, Laboratorio de Enfermedades Tropicales, Campeche, Campeche, Mexico; 2 Universidad Nacional Autónoma de México, Facultad de Medicina, Unidad de Investigación en Medicina Experimental, Ciudad de México, Mexico; 3 Universidad Autónoma de Campeche, Centro de Investigaciones Biomédicas, Laboratorio de Enfermedades Transmitidas por Vectores y Zoonosis, Campeche, Campeche, Mexico

**Keywords:** Leishmania, Cutaneous leishmaniasis, Ear, Urban area, Vector-borne emerging disease

## Abstract

Cutaneous leishmaniasis represents 99% of all reported leishmaniasis cases in Mexico and typically occurs in agricultural or sylvatic areas. Campeche State is endemic for leishmaniasis; however, there are no previous records of urban *Leishmania* transmission. This report presents a case of cutaneous leishmaniasis in a 75-year-old man residing in an urban area. The patient presented with a three-month-old lesion on the right ear following an initial misdiagnosis of a bacterial infection. Given the suspicion of leishmaniasis, a tissue imprint was collected, revealing the presence of *Leishmania* amastigotes. Subsequently, amplification and sequencing of the Alanine aminotransferase and Internal transcribed spacer subunit 1 genes confirmed the presence of *Leishmania mexicana*. The patient was then treated with intralesional meglumine antimoniate. This case is significant as it marks the first confirmed human transmission of *L. mexicana* in an urban environment in Campeche State, demonstrating the importance of considering this pathology in patients with skin lesions originating from non-endemic areas in Mexico.

## INTRODUCTION

Leishmaniasis is a neglected parasitic disease primarily found in tropical and subtropical regions of the world. It manifests in three primary clinical forms: localized cutaneous leishmaniasis, mucocutaneous leishmaniasis, and visceral leishmaniasis^1^. The causative agents are flagellate parasites belonging to the genus *Leishmania*, transmitted via the bites of *Lutzomyia* or *Phlebotomus* sandflies. Globally, it is estimated that more than 12 million people are currently infected, with an additional 350 million at risk of infection. In Latin America, cases of leishmaniasis have been reported in urban areas of countries such as Colombia^2^, Venezuela^3^, and Brazil^4^.

In Mexico, endemic areas with the highest prevalence of leishmaniasis infection include Campeche State, Tabasco State, and Quintana Roo State^5^. Within Campeche State, the municipalities with the highest number of leishmaniasis cases include Hopelchen municipality, Calakmul municipality, and Candelaria municipality. The earliest records of leishmaniasis in this region date back to the 1943 “chewing gum” boom, during which individuals extracting resin from chico zapote trees were frequently exposed to vectors within the sylvatic cycle. Subsequently, as the “chewing gum” industry declined, a shift towards agricultural activities occurred, leading to a rural transmission cycle predominantly affecting populations near agricultural fields, including coffee and cocoa plantations^6^.

In the Campeche State, the deforestation of sylvatic areas for new agricultural lands and infrastructure projects, such as the “Mayan train,” has recently led to an increase in *Leishmania* infections. However, there are no published records of naturally transmitted cutaneous leishmaniasis in urban areas of Campeche. In this paper, we describe the first report of a case of natural transmission of urban leishmaniasis in Escarcega municipality, Campeche State, Mexico, and the molecular identification of the causative agent.

## CASE REPORT

**Figure 1:**
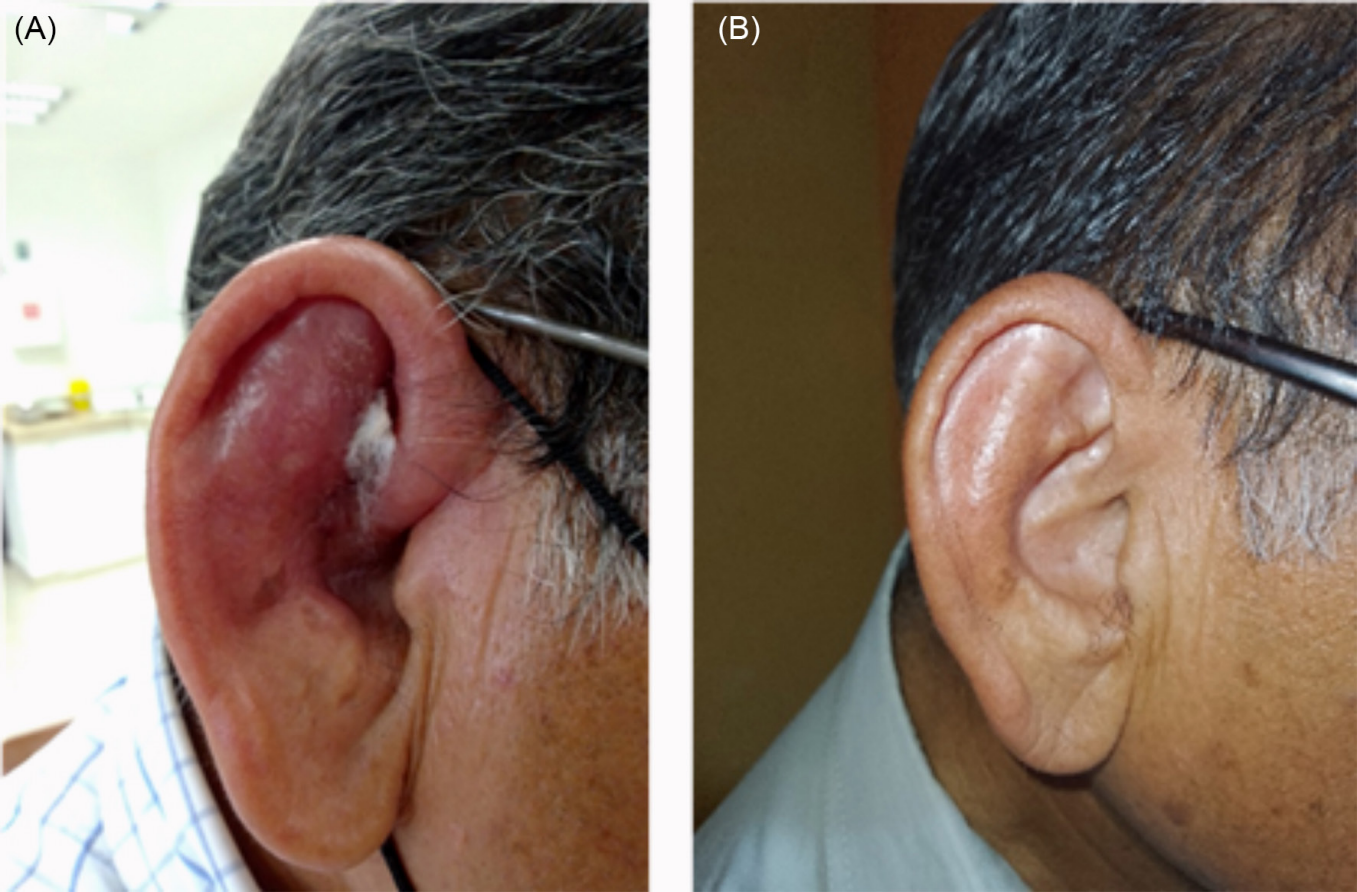
(A) Cutaneous lesion on the right ear; (B) Ear after treatment.

On August 30, 2022, a 70-year-old male patient from Escarcega municipality (18°36’40.9” N 90°44’29.4” W), Campeche State, visited the Biomedical Research Center of the Autonomous University of Campeche with a three-month-old lesion on his right ear (Figure 1A). During the interview, the patient—retired and engaged in household chores—reported no relevant medical history, medication use, alcohol consumption, or damage to the injury site. He mentioned maintaining fruit trees in his residence located in the central area of the Escarcega municipality. Over the past eight months, his travel was limited to the state capital, with no visits to sylvatic sites, which are potential risk areas for *Leishmania* infection. He also mentioned the presence of domestic dogs, opossums, and wild squirrels on his property.

The patient described the lesion as beginning with an itchy welt that progressively grew, became inflamed, and eventually ulcerated his ear. Despite initial treatment with intramuscular antibiotics for approximately two months by his primary physician, there was no improvement. Subsequently, upon seeking a second opinion in the capital, the new physician suspected a potential case of leishmaniasis. The patient was referred to the Biomedical Research Center of the Autonomous University of Campeche for further investigation.

**Figure 2:**
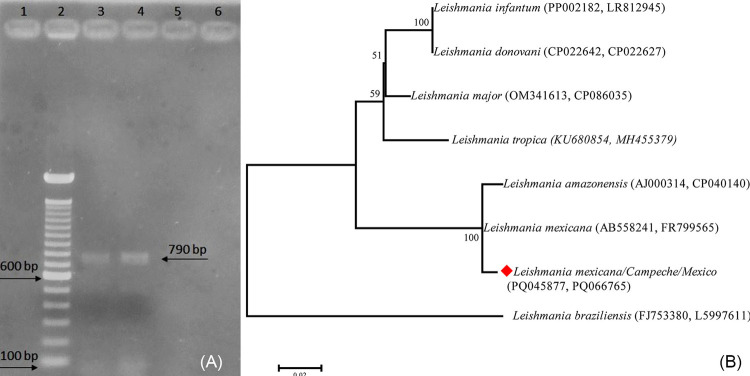
Molecular identification of Leishmania parasite: (A) Agarose gel (2%): Lane 1, Water (reagent control); Lane 2, 100-bp molecular weight marker; Lane 3, Positive control, *Leishmania mexicana*; Lane 4, Patient sample; (B) Maximum likelihood phylogenetic tree generated with 833 bp of concatenated fragments of the ITS-1 (321 bp) and ALAT (512 bp) genes using the Hasegawa-Kishino-Yano (HKY) substitution model with Gamma distribution (+G). (InL= −1989.952).

A tissue imprint was collected from the edge of the lesion and stained with Giemsa for microscopic examination using a 100× immersion objective. The staining showed rounded forms with a nucleus and kinetoplast consistent with *Leishmania* amastigotes. RPMI-1640 (Gibco, UK) culture media were inoculated with aspirate from the lesion, resulting in the growth of *Leishmania* promastigotes. DNA extraction was performed using a commercial kit (DNeasy^®^ Blood & Tissue Kit, QIAGEN, Germany) for identification via polymerase chain reaction (PCR) testing. The oligonucleotides IR1 (5’-GCTGTAGGTGAACCTGCAGCAGCTGG ATCATT-3’)^7^ and LM17 (5’-CCCCTCTCCTCCTCCCC-3’)^8^ successfully amplified a 790 base pairs (bp) product specific to *Leishmania mexicana* (Figure 2A). Oligonucleotides specific for *Leishmania braziliensis* LM9 (5’-GGACGAGCTCATGGCGCC-3’) and LV2 (5’-CAATGCAGTCATCCTTTC-3’) did not yield any amplification product. Moreover, parasite typing was conducted using PCR to amplify and sequence a 589 bp fragment of the alanine aminotransferase gene (ALAT) and a 300 bp fragment of the internal transcribed spacer 1 (ITS1), following procedures described by Fernández-Figueroa *et al.* ^9^. The positive PCR products were purified by Macrogen, Korea. The sequences obtained in this study were deposited in GenBank under accession Nº PQ066765 and PQ045877 and compared with existing GenBank entries using the BLASTn tool. Additionally, a phylogenetic reconstruction was conducted by concatenating both genes and using the Maximum Likelihood method with 10,000 bootstrap replicates in MEGA 11.0. Blast analysis of the sequences from the ITS1 and ALAT genes identified *L. mexicana*, with a similarity of 98% (315/320 bp) and 99% (510/511 bp), respectively, compared to GenBank accession Nº AB558241 and XM_003873118. The phylogenetic analysis confirmed the presence of *L. mexicana*, clustering our sequences with reference sequences of *L. mexicana* and *L. amazonensis* deposited in GenBank, with a support value of 100 (Figure 2B).

Following confirmation of the diagnosis of localized cutaneous leishmaniasis, treatment was initiated with meglumine antimoniate (Glucantime^®^, Aventis, Brazil), administered intralesionally at one ampule injected per week for five weeks. By the end of this treatment regimen, complete resolution of the lesion was observed (Figure 1B).

The patient signed an informed consent form, and confidentiality was strictly maintained. This study was approved by the Research Ethics Committee of the Campeche Secretary of Health (CEI30042024/A2).

## DISCUSSION

In Mexico, localized cutaneous leishmaniasis is the most frequently reported clinical form among individuals infected with parasites of the genus *Leishmania* ^10^. This condition primarily affects individuals who interact with the vectors in sylvatic and rural settings. The significance of this case is attributed to the patient acquiring the infection in an urbanized environment, as epidemiologically confirmed by no history of visiting endemic or high-risk areas for infection. However, the increasing presence of sylvatic animals such as squirrels and opossums due to habitat destruction and other environmental factors likely contributes to the establishment of an urban transmission cycle of leishmaniasis^11^. A recent systematic review identified deforestation, primarily due to road and railroad construction and the establishment of new human settlements, as a primary risk factor for urban expansion of leishmaniasis^12^.

Despite Campeche State being one of the states with the highest prevalence of leishmaniasis, some cases are not promptly and accurately diagnosed, creating a worrisome scenario from a public health perspective^13^. In this case, the patient received approximately two months of antibiotic treatment for a presumed bacterial infection, revealing that certain physicians may overlook this neglected tropical zoonosis as a possible etiology for ulcerated lesions. Leishmaniasis, often perceived as occurring exclusively in rural areas, likely contributes to misdiagnoses in the Campeche State. A similar challenge has been reported in the United States, where medical practitioners are unfamiliar with leishmaniasis, compounded by the medical literature predominantly associating it with tropical regions, potentially leading to underdiagnosis^14^. In Campeche State, the Ministry of Health clinically diagnoses leishmaniasis and confirms it via lesion imprints, whereas species identification is primarily conducted by research centers. Identifying *Leishmania* species accurately is crucial for appropriate treatment^15^. Previous studies have shown that sequencing and phylogenetic analysis of both molecular markers enable accurate identification of infecting *Leishmania* species in patient isolates and biological samples from wildlife^16^. In this case, the species identified was *L. mexicana*, which is recognized as the primary causative agent of localized cutaneous leishmaniasis, aligning with previous reports. However, *L. braziliensis*, another causative agent of localized cutaneous leishmaniasis^15^, was not detected in this case. Characterizing the *Leishmania* species in this study contributes to understanding the species circulating in emerging areas of transmission.

Climate change, deforestation, and the destruction of sylvatic areas due to the expansion of urban settlements and roads have increased human contact with vectors and reservoirs of the *Leishmania* parasite, leading to an increase in localized cutaneous leishmaniasis cases and the first documented case of urban leishmaniasis caused by *L. mexicana* in the Campeche State. This case could be indicative of the establishment of an urban transmission cycle for leishmaniasis. The presence of urban leishmaniasis in the state is concerning as it may suggest the emergence of a previously unrecognized transmission cycle, in which the vectors and reservoir hosts facilitating transmission remain unidentified. Studies in the region have identified 21 species of sandflies, including *Bichromomyia olmeca olmeca, Lutzomyia cruciate*, and *Psathyromyia shannoni*, which have been implicated as vectors of *L. mexicana* ^17,18^. Additionally, state records report *Peromyscus yucatanicus* and *Heteromys gaumeri*, both rodent species recently identified as reservoir hosts in new transmission foci of leishmaniasis in the neighboring Yucatan State^19,20^. Therefore, research to understand the parasite’s transmission cycle in urban conditions in the Campeche State should be prioritized.

## CONCLUSION

Physicians must recognize that leishmaniasis is not restricted to sylvatic and rural areas but can also occur in urban environments. Public awareness campaigns are essential to engage the population in the prevention of leishmaniasis.
